# Selective Inhibition of the ABCG2 Transporter by Primaquine Derivatives Reverses the Multidrug Resistance of Tumor Cells

**DOI:** 10.3390/ijms26115367

**Published:** 2025-06-03

**Authors:** Marija Mioč, Maja Beus, Karla Carević, Zrinka Rajić, Balázs Sarkadi, Ágnes Telbisz, Marijeta Kralj

**Affiliations:** 1 Division of Molecular Medicine, Ruđer Bošković Institute, Bijenička c. 54, 10000 Zagreb, Croatia; marija.mioc@irb.hr (M.M.); kcarevic4@gmail.com (K.C.); 2 Department of Medicinal Chemistry, University of Zagreb Faculty of Pharmacy and Biochemistry, A. Kovačića 1, 10000 Zagreb, Croatia; maja.beus@duke.edu (M.B.); zrajic@pharma.hr (Z.R.); 3 Institute of Molecular Life Sciences, HUN-REN Research Centre for Natural Sciences, Magyar Tudósok krt. 2, 1117 Budapest, Hungary; sarkadi.balazs@ttk.hu

**Keywords:** ATP-binding cassette (ABC) transporters, ABCG2, P-glycoprotein, multidrug resistance, primaquine derivatives, in vitro functional studies

## Abstract

Multidrug resistance (MDR) poses a significant challenge in cancer therapy, often leading to treatment failure and relapse. ATP-binding cassette (ABC) transporters, particularly ABCG2, play a pivotal role in MDR development by actively expelling chemotherapeutic agents from cancer cells. This study investigates the effects of two groups of primaquine derivatives—fumardiamides (**1a**–**d**) and *bis*-ureas (**2a**, **b**), both bearing halogenated benzene rings—on the activity of P-glycoprotein (P-gp) and ABCG2. Their potential to reverse MDR was evaluated through a series of functional assays aimed at comparing transporter–compound interactions. The results indicated that fumardiamide derivatives, specifically **1a**, **1b**, and **1d**, exhibited potent inhibition of ABCG2 while having no effect on P-gp, demonstrating a selective mode of action. The tested derivatives displayed low to moderate cytotoxicity and did not affect ABCG2 expression or localization. Moreover, these compounds enhanced the sensitivity of drug-resistant cancer cell lines to mitoxantrone, underscoring their potential to overcome ABCG2-mediated MDR. These findings suggest that chemical modifications of primaquine, particularly the incorporation of fumardiamide moieties, confer novel biological properties, providing promising leads for the development of selective ABCG2 inhibitors.

## 1. Introduction

Chemotherapy is a key component of cancer treatment, alongside surgery, immunotherapy, and radiotherapy. However, in many cases, intrinsic or acquired resistance to chemotherapy complicates treatment. The development of so-called multidrug resistance (MDR) in response to cancer chemotherapy represents one of the major challenges in clinical practice, often leading to treatment failure and disease relapse [1,2,3]. MDR is caused by the presence of promiscuous membrane transporters belonging to the ATP-binding cassette (ABC) transporter family. These transporters are capable of effluxing a wide variety of chemically unrelated drugs. MDR is particularly common in cancers originating from tissues in which these transporters are naturally highly expressed, such as epithelial cells in the kidney, liver, and colon. However, the overexpression of ABC transporters can also be acquired as a consequence of chemotherapy treatment [4].

ABC multidrug transporters, such as P-glycoprotein (P-gp, ABCB1) and breast cancer resistance protein (BCRP, ABCG2), are expressed in various tissues and physiological barriers, where they play a key protective role by limiting the accumulation of xenobiotics. Due to their overlapping substrate specificities, these transporters often act cooperatively to efflux a broad spectrum of hydrophobic and charged compounds, including many clinically relevant drugs and toxins. Their expression at critical barriers—such as the blood–brain, blood–testis, and maternal–fetal barriers—as well as in peripheral tissues, has a significant impact on drug pharmacokinetics and therapeutic efficacy [5,6,7]. In addition, ABCG2 is frequently expressed in stem cells, where it plays a crucial role in maintaining their phenotype and protecting them from xenobiotics and other toxins in vivo [8]. Cancer relapse is often driven by a small population of drug-resistant cells [9,10,11], which share many characteristics with stem cells and are referred to as cancer stem-like cells (CSCs). CSCs possess the capacity for self-renewal and can initiate the formation of new tumors [12,13,14]. In many cases, CSCs express specific surface markers distinct from those of the surrounding tumor cells, including the ABC transporters ABCB1 and/or ABCG2. Both experimental and clinical studies have shown that CSCs can survive treatments that eliminate the majority of tumor cells. This resistance is largely attributed to the presence of MDR proteins on their surface. Consequently, modulating the activity or expression of these transporters is of great clinical importance for overcoming MDR and improving the efficacy of anticancer therapies [15,16].

To date, many pharmacological agents have been discovered as potent inhibitors of P-gp and ABCG2, but none of them have been clinically approved due to clinical inefficacy, pharmacokinetic interactions, and safety concerns [7,17,18,19]. Ongoing research on the development of new MDR inhibitors, therefore, focuses on achieving greater inhibitory potency, minimizing general cytotoxicity, and increasing selectivity toward individual ABC transporters in order to reduce side effects [20].

One of the key strategies involves screening compounds using structure–activity relationship (SAR) models to predict how specific chemical motifs influence the binding of small molecules to protein structures at defined inhibitory sites [3,21,22]. Another important strategy is drug repurposing, in which approved drugs are used for new therapeutic purposes. This approach offers several advantages, including reduced costs, a lower risk of failure, and a shorter time to market. For example, drugs such as calcium-channel blockers, anti-HIV drugs, and tyrosine kinase inhibitors have already been identified as potent ABCG2 inhibitors [22].

Certain clinically approved antimalarial drugs have been shown to exert anti-tumor effects [23,24,25,26,27,28]. Various chemical classes of antimalarials exhibit anticancer activity, can reverse tumor cell resistance to treatment and inhibit the activity of P-gp and/or ABCG2. The inhibition of MDR transporters likely contributes to reducing drug resistance [29,30,31,32].

We combine two complementary strategies in the search for a drug with enhanced anti-tumor activity: chemical modification of an approved antimalarial drug and the investigation of its interaction with MDR transporters. Chemical modifications of registered antimalarials—particularly hybridization with various molecular scaffolds—have also been explored in other studies as a promising approach for the development of novel bioactive agents with diverse biological profiles and/or reduced toxicity [32,33]. In this study, we examined the effects of two groups of primaquine derivatives bearing halogenated benzene rings—fumardiamides (**1a**–**d**) and *bis*-ureas (**2a**, **b**)—on the activity and expression of P-gp and ABCG2 (see Table 1). The anti-tumor activity of these compounds has already been demonstrated [34,35].

By using various in vitro cellular and membrane vesicular assays, we have shown that some of the primaquine fumardiamides have potent inhibitory effects on ABCG2 and can sensitize cancer cell lines to the chemotherapeutic agent mitoxantrone. We have also shown that these compounds act as substrates of the P-gp transporter, they do not inhibit P-gp activity and are thus selective inhibitors of ABCG2. These data suggest that primaquine derivatives have the potential to be used in cancer therapy to reverse ABCG2-mediated MDR.

## 2. Results

### 2.1. Effect of Primaquine Derivatives on ABCG2 Transporter’s Activity

#### 2.1.1. Primaquine Derivatives Show Low Cytotoxicity Against Parental and ABCG2 Transporter-Overexpressing Model Cell Lines

To test the cytotoxicity of primaquine fumardiamides **1a**–**d**, as well as the *bis*-urea primaquine derivatives **2a** and **2b** in PLB/ABCG2 and the parental PLB-985 cell line, we used the MTT assay. Fumardiamides with *para*-halogenated benzene ring **1b** and **1d** showed no significant toxicity in either cell line, whereas their *meta*-substituted analogs **1a** and **1c** were more toxic. *Bis*-urea **2b** with a trifluoromethyl substituent in the *para* position was the most toxic among the tested derivatives, with the same effect in both cell lines. In general, all primaquine derivatives had the same effect in both the parental and ABCG2-overexpressing cell lines, with the exception of **1c** and **2a**, which showed weaker antiproliferative effects against the PLB/ABCG2 cell line than the parental PLB-985 (Table 1).

#### 2.1.2. Primaquine Derivatives Inhibit Hoechst 33342 Efflux in Model Cell Lines

**Figure 1:**
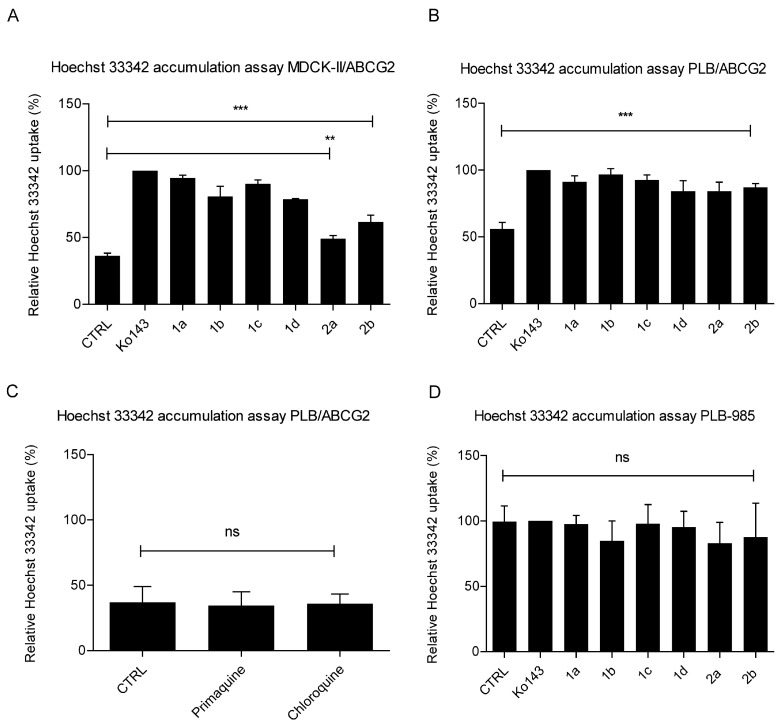
Inhibition of Hoechst 33342 transport in MDCK-II/ABCG2, PLB/ABCG2, and PLB-985 cell lines via primaquine derivatives **1a**–**d** and **2a**, **b** (**A**,**B**,**D**) and conventional anti-malarial drugs primaquine and chloroquine in PLB/ABCG2 line (**C**). Hoechst 33342 is non-fluorescent outside of cells and only shows fluorescence when taken up by cells, binding to nuclei. Uptake depends on ABCG2 activity. The mean fluorescence intensity of the sample was measured 60 min after the addition of 0.5 µM of Hoechst via a microplate fluorometer. Maximum (100%) uptake was determined through the addition of 1 µM Ko143 (known ABCG2 inhibitor), and the effect of the test compounds (applied at 10 µM) was expressed relative to this data. Control samples (CTRL) were untreated. Data are shown as means ± SDs of three independent experiments. One-way ANOVA with Dunnett’s post hoc test was used for statistical analysis (** *p* < 0.01; *** *p* < 0.001; ns—not significant).

To test the inhibitory effect of primaquine derivatives on the ABCG2 transporter, we first applied the Hoechst 33342 uptake assay with cells overexpressing ABCG2 (MDCK-II/ABCG2, PLB/ABCG2) and with parental cells lacking ABCG2 expression (MDCK-II, PLB-985). This assay is based on the accumulation of the fluorescent dye Hoechst 33342 into the cells and its active extrusion via the ABCG2 transporter. In the presence of a reference inhibitor or an inhibitory compound, the efflux of the fluorescent dye is reduced, resulting in increased fluorescence within the cells, which can be measured. Figure 1A–D shows that the tested primaquine derivatives exhibit inhibitory potential towards ABCG2 in the MDCK-II/ABCG2 and PLB/ABCG2 cell lines. When we performed the assay with the parental cell lines, there was no change in fluorescence after the application of the reference inhibitor Ko143 or the primaquine derivatives, as the dye continuously accumulates in the cells due to the lack of the ABCG2 transporter (Figure 1D). We also tested the parent drug primaquine and the structurally related antimalarial drug chloroquine, and neither of them showed an inhibitory effect on ABCG2 (Figure 1C).

**Figure 2:**
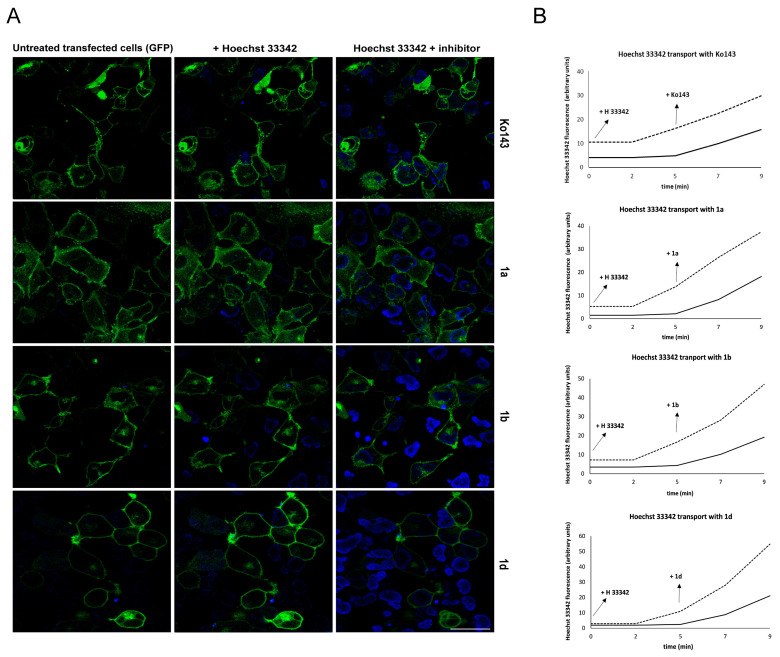
Real-time transport of Hoechst 33342 in the HEK 293 cell line transfected with the EGFP-ABCG2 vector (EGFP-G2 HEK 293). Dye uptake was monitored using confocal microscopy before and after the addition of inhibitors. (**A**) The left column shows cells before any treatment. Transfected cells expressing GFP-tagged ABCG2 transporter (green) shows green membrane staining while non-transfected cells are not visible. The middle column shows the Hoechst 33342 (blue) staining of nuclei. This appears only in non-transfected cells after the addition of Hoechst 33342. The right column shows Hoechst 33342 staining (blue) and EGFP labeling (green) in the cells after the addition of inhibitor compounds labeled for each row. Both transfected (EGFP-green) and non-transfected (dark) cells showed blue staining after the addition of the inhibitory compound (Ko143, **1a**, **1b**, or **1d**). The bar represents 25 μm. (**B**) Hoechst staining intensity quantification plots for each compound in transfected (solid lines) and non-transfected (dashed lines) cells. At least 50 cells were analyzed at time points 0 min (Hoechst dye added), 2 min, 5 min (inhibitor added), 7 min, and 9 min.

To follow the uptake of Hoechst 33342 in real time, we used the HEK-293 cell line, transfected it with a vector carrying the EGFP-tagged ABCG2 protein, and followed the procedure described in Orbán et al. [36]. We analyzed two groups of cells based on green fluorescence: with green fluorescence in the plasma membrane (transfected) and without (non-transfected). Forty-eight hours after transfection, we examined the accumulation of blue Hoechst 33342 fluorescence in the nucleus of each group of cells using confocal microscopy and quantified the Hoechst 33342 uptake from the acquired images. Figure 2A—(middle column, B) shows that Hoechst 33342 rapidly accumulates in non-transfected cells, whereas transfected cells do not show blue-stained nuclei. After five minutes of Hoechst 33342 efflux, upon the addition of the reference inhibitor Ko143 or primaquine derivatives **1a**, **1b**, or **1d**, we were also able to detect blue staining in transfected cells, which is due to the inhibition of ABCG2 via these compounds (Figure 2A—right column, B). At the same time, the addition of the inhibitory compound had no effect on the already higher accumulation rate of blue fluorescence in non-transfected cells (Figure 2B).

#### 2.1.3. Primaquine Fumardiamides Show Best Inhibitory Potential

**Figure 3:**
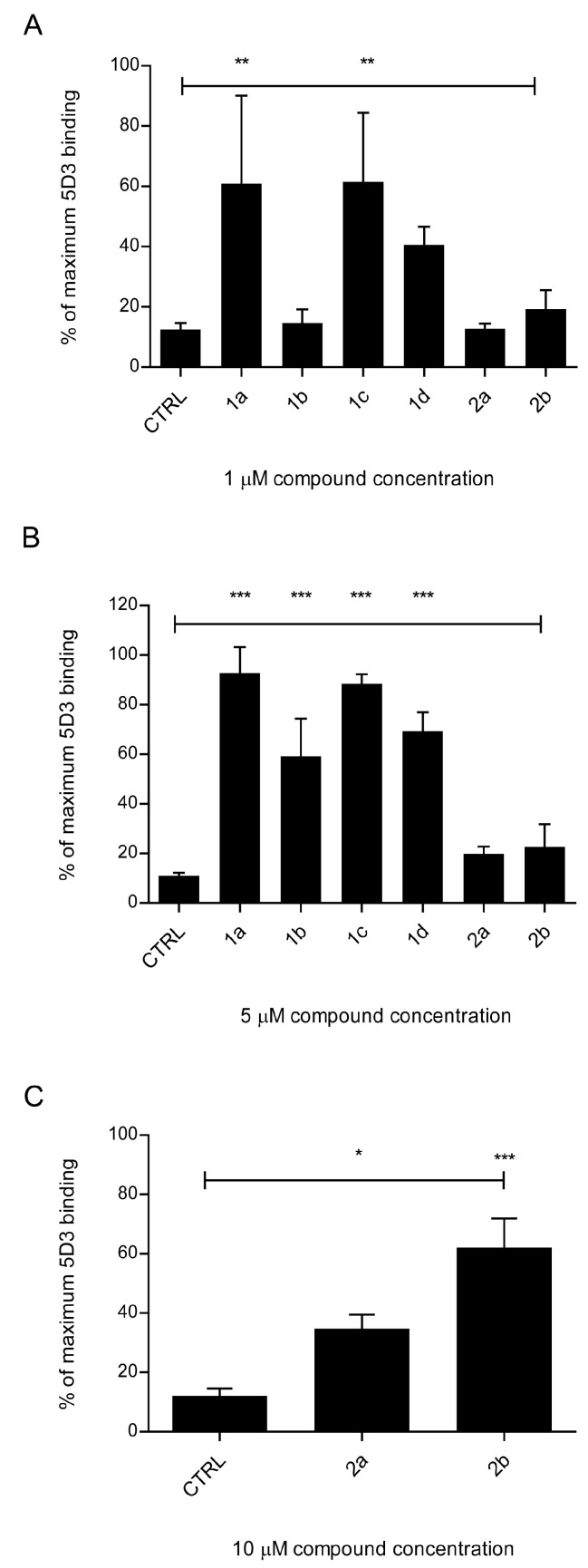
A 5D3 antibody binding shift assay in PLB/ABCG2 cells measured via flow cytometry; 5D3 is a conformation-sensitive antibody against ABCG2. Cells were treated with 1 µM (**A**), 5 µM (**B**), or 10 µM (**C**) primaquine derivatives **1a**–**d** and **2a**, **b** prior to binding of the fluorescently labeled 5D3 antibody. The mean fluorescence intensity was measured and is expressed as a percentage of the maximum 5D3 binding in the presence of 1 µM Ko143. Data are expressed as means ± SDs of three independent experiments. One-way ANOVA with Dunnett’s post hoc test was used for statistical analysis (* *p* < 0.05; ** *p* < 0.01; *** *p* < 0.001).

The test for fluorescent substrate uptake gives us information about the interaction of the compounds with a transporter, but tells us little about the nature of this interaction. To test whether the compounds have a strong inhibitory effect on ABCG2, we performed the 5D3 antibody binding shift assay. This antibody binds to a certain conformation related to the substrate binding state of ABCG2. This phenomenon is most observable with inhibitor-type substrates. Previously, it was shown that inhibitors cause a concentration-dependent elevation of 5D3 binding on ABCG2-expressing cells, which can be seen as a fluorescence shift in the flow cytometry histogram with fluorescently labeled 5D3 antibody [37,38,39]. We tested the inhibitory activity of our primaquine derivatives at concentrations of 1 and 5 µM and expressed their activity as a percentage of maximal 5D3 binding, which corresponds to maximal ABCG2 inhibition with 1 µM Ko143. All fumardiamides **1a**–**d**, at a concentration of 5 µM, caused a significant shift in mean fluorescence intensity compared to Ko143, which indicates an inhibitor-type interaction with ABCG2. Two of them with *meta*-substituted benzene rings, namely **1a** and **1c**, showed 60% maximal inhibition at 1 µM (Figure 3A,B). *Bis*-ureas **2a** and **2b** showed no inhibitory effect at 1 and 5 µM concentrations, so we tested them at a higher concentration (10 µM). Trifluoro-derivative **2b** showed more than 60% maximal inhibition, whereas chloro-derivative **2a** showed less than 40%, even at the high concentration (Figure 3C).

#### 2.1.4. Primaquine Derivatives Inhibit the ATPase Activity of the ABCG2 Transporter

To confirm the inhibitory effect of the tested substances, we performed the ATPase assay using inside-out membrane vesicles prepared from the human ABCG2-expressing Sf9 cell culture [40]. This assay allows the measurement of the transporter’s activity based on drug transport-coupled ATPase activity. All ABC transporters utilize the energy of ATP for substrate transport; inorganic phosphate is released in each transport cycle, which can be measured by a simple colorimetric reaction. ABC multidrug transporters have a drug-independent basal (uncoupled) ATPase activity that is modified quantitatively by drug interactions (transport-coupled ATPase activity). Inhibitory compounds slow down or block the transport cycle, leading to a decrease in ATPase activity, while actively transported substrates stimulate it in most cases [41].

**Figure 4:**
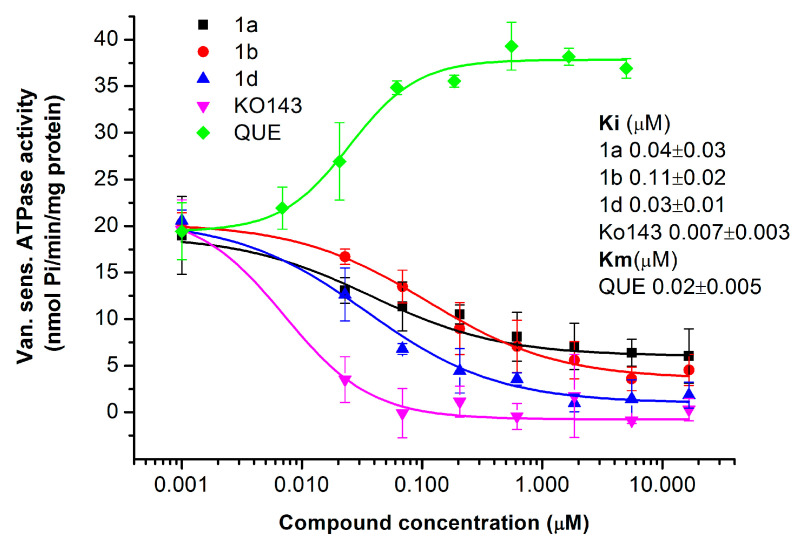
Impact of primaquine fumardiamide derivatives **1a**, **1b**, and **1d** on vanadate-sensitive ATPase activity of ABCG2 transporter. The ATPase activity was measured in inside-out membrane vesicles with overexpression of ABCG2. The effect of the compounds was compared with that of Ko143 and quercetin (QUE) as reference compounds. The ATPase activity was measured with a colorimetric assay that measures liberated phosphate (Pi). Data in the graphs show the average of three independent experiments ± SD performed in triplicate. Ki (inhibitory constant) and Km (Michaelis-Menten) constants presented in the picture were determined by Hill1 fitting.

We tested the effect of primaquine fumardiamide derivatives **1a**, **1b**, and **1d** on the ATPase activity of the ABCG2 transporter in a wide concentration range (Figure 4). Comparison of Michaelis–Menten constants (Hill-1 fitting) of ATPase activity shows that derivatives **1a**, **1b**, and **1d** exerted similar inhibitory interactions (approximate Ki were 0.04, 0.11, and 0.03 µM, respectively). However, the Ki of the known specific ABCG2 inhibitor Ko143 was significantly less at 0.007 µM. In contrast, the actively transported substrate quercetin stimulated the ATPase activity with a Km of 0.02 μM.

#### 2.1.5. Drug-Resistant Model Cell Line Expressing ABCG2 Is Sensitized to the Chemotherapeutic Agent Mitoxantrone with the Primaquine Derivatives **1a**, **1b**, and **1d**

Model cell line overexpressing the ABCG2 protein (PLB/ABCG2) shows increased resistance to the conventional chemotherapeutic agent mitoxantrone, which is a substrate of ABCG2, compared to its parental counterpart, PLB-985 cell line (Table 2). We tested the ability of compounds **1a**, **1b**, and **1d** to reverse the resistance phenotype of the PLB/ABCG2 cell line to mitoxantrone. Mitoxantrone toxicity was measured with an MTT assay in a serial dilution of mitoxantrone applied together with 0.5 µM of the investigated compounds. The parental PLB-985 cell line showed high sensitivity to mitoxantrone compared to the resistant counterpart, but no further changes were observed in the presence of the investigated compounds (Table 2). On the other hand, all three compounds successfully sensitized the more resistant PLB/ABCG2 cell line to mitoxantrone and shifted its IC_50_ value from 0.12 to 0.01 µM (**1a** and **1d**) or 0.02 µM (**1b**). This effect is similar to the effect of the ABCG2 inhibitor Ko143, which shifted the IC_50_ of MX to 0.01 µM (Table 2).

#### 2.1.6. Primaquine Derivatives Have No Effect on the Expression or Localization of the ABCG2 Transporter

**Figure 5:**
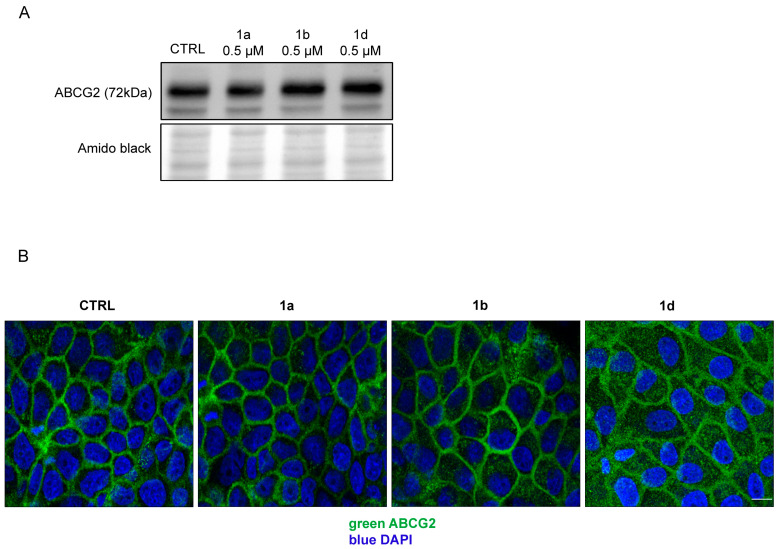
Impact of the selected primaquine derivatives on ABCG2 expression and localization in the plasma membrane. (**A**) PLB/ABCG2 cell line was treated with 0.5 µM **1a**, **1b**, or **1d** for 72 h, and protein expression was detected with the Western blot method using anti-ABCG2 antibody BXP-21. Amido black staining was used as a loading control. (**B**) Localization of ABCG2 in MDCK-II/ABCG2 line in control and inhibitor (5 µM primaquine derivatives, 72 h) treated samples. Immunofluorescence staining was performed with fluorescently labeled anti-ABCG2 antibody (BXP-21, green). Nuclei were stained with DAPI. The bar represents 8 µm.

To show that ABCG2 levels and localization are not altered by these treatments, we used the Western blot and immunolocalization method. None of the compounds altered ABCG2 quantity in PLB/ABCG2 cells after 72 h of treatments with **1a**, **1b**, and **1d** (Figure 5A). Localization of the ABCG2 transporter was investigated in the MDCK-II/ABCG2 cell line via immunofluorescence. Figure 5B shows normal cell membrane localization of ABCG2 even after 72 h of treatment with the tested compounds.

### 2.2. Effect of Primaquine Derivatives on the Activity of the P-gp Transporter

#### 2.2.1. The P-gp Transporter-Overexpressing Cell Line Is More Resistant to Primaquine Fumardiamides **1a**, **1b**, and **1d** Compared to the Parental Cell Line

ABC multidrug transporters have overlapping substrate specificity; therefore, some compounds can act as dual inhibitors of two different ABC transporters or even as pan-inhibitors, which is rarely reported [22]. The most active primaquine derivatives **1a**, **1b**, and **1d** were tested on another important transporter involved in the MDR phenotype, P-gp. First, we tested the cytotoxicity of these compounds in the P-gp-overexpressing cell line A2780/Adr and the parental cell line A2780 using the MTT assay. Compounds **1b** and **1d** were non-toxic at relevant concentrations, whereas compound **1a** exhibited mild toxicity, with the parental A2780 cell line being slightly more sensitive (Table 3).

#### 2.2.2. Primaquine Derivatives **1a**, **1b**, and **1d** Have No Effect on Substrate Extrusion Mediated via the P-gp Transporter

**Figure 6:**
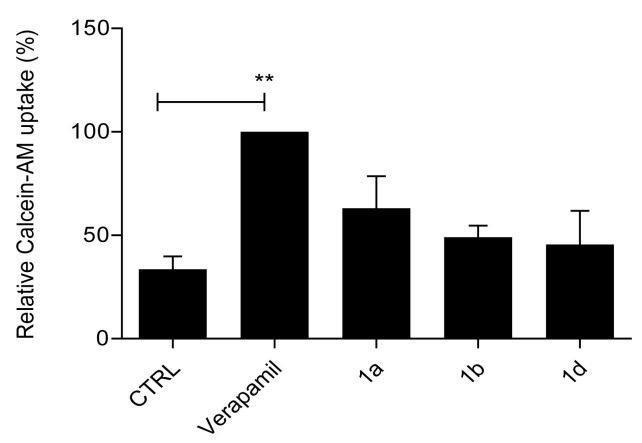
Inhibition of calcein-AM accumulation in the cell line A2780/Adr (P-gp expressing) via the primaquine derivatives **1a**, **1b**, and **1d**. The mean fluorescence intensity of calcein was measured after treatment with 10 µM of the compounds. Calcein-AM uptake is shown relative to maximal uptake when P-gp was inhibited by 10 µM of verapamil. CTRL is an untreated sample with active P-gp. Data are expressed as means ± SDs of three independent experiments. One-way ANOVA with Tukey’s post hoc test was used for statistical analysis ** *p* < 0.01).

To test whether compounds **1a**, **1b**, and **1d** have an inhibitory effect on the P-gp transporter, we performed the calcein–AM uptake assay with cells overexpressing P-gp (A2780/Adr). The assay is similar to the H-33342 accumulation assay, in which the efflux of the fluorescent dye calcein is reduced in the presence of the P-gp inhibitory compound, as evidenced by increased fluorescence within the cells. As can be seen in Figure 6, none of the compounds had a significant effect compared to the known P-gp inhibitor verapamil, indicating that they do not inhibit the P-gp transporter.

#### 2.2.3. The Primaquine Derivatives **1a**, **1b**, and **1d** Stimulate the Activity of the P-gp Transporter

**Figure 7:**
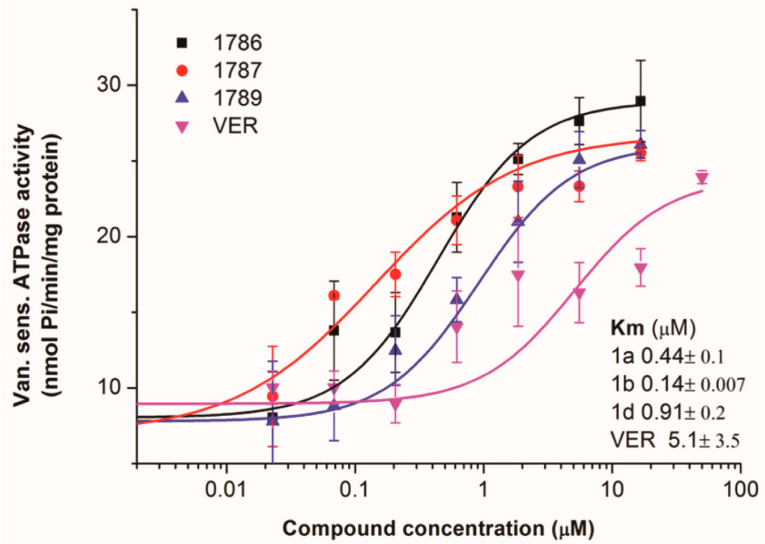
Impact of the primaquine derivatives **1a**, **1b**, and **1d** on the vanadate-sensitive ATPase activity of the P-gp transporter. ATPase activity was measured in inside-out membrane vesicles with overexpression of the P-gp transporter. The ATPase activity was measured using a colorimetric assay that measures liberated phosphate (Pi). The effect of the compounds was compared with that of verapamil (VER). The data in the graphs show the average of three independent experiments ± SD performed in triplicate. Km constants presented in the picture were determined by Hill1 fitting.

Interaction between compounds **1a**, **1b**, and **1d** and the P-gp transporter was characterized by the sensitive ATPase assay in Sf9 membrane vesicles containing human P-gp prepared similarly to ABCG2 membranes. Figure 7 shows that all the tested compounds strongly stimulate the ATPase activity of P-gp, similarly to verapamil [42]. All three compounds, **1a**, **1b**, and **1d**, had a stronger ATPase stimulatory effect than verapamil (Michaelis-Menten constants K_m_ were 0.44, 0.14, 0.91, and 5.1 µM, respectively). These results suggest that, in micromolar concentrations, the selected primaquine derivatives are probably transported as P-gp substrates without significant inhibitory activity. Overall, the obtained results indicate that the tested primaquine derivatives are selective ABCG2 inhibitors.

## 3. Discussion

ABC transporters, with their overlapping substrate specificities, effectively protect the body against various toxic drugs, which is a vital function. Their expression levels can influence drug pharmacokinetics, while drug–drug interactions can also modulate transporter activity [4]. The strong inhibition of all ABC transporters can lead to serious side effects due to the increased toxicity of several compounds resulting from their accumulation in our bodies [43]. Therefore, the investigation of novel ABC transporter inhibitors requires knowledge of the expression level of a particular transporter and its interaction with the specific anticancer drug to be used in combination with this inhibitor to improve drug efficacy [4,44]. To avoid side effects, an ABC-selective compound might have a better therapeutic benefit. Since cancer cells can acquire MDR phenotype by expressing single or multiple types of ABC transporters, as is the case with CSCs, careful selection between specific and pan-inhibitors may be useful in preventing cancer recurrence [22]. The three most important characteristics of a good ABC transporter inhibitor are, therefore, low cytotoxicity, high inhibitory power, and high selectivity for one specific multidrug transporter [20,22].

In this study, we investigated two groups of primaquine-derived compounds—fumardiamides (**1a**–**d**) and *bis*-ureas (**2a**, **b**)—and their ability to inhibit ABCG2 and/or P-gp transporter activity. These compounds were investigated in order to find an efficient, non-toxic, ABCG2-selective compound for further development. It was previously shown that the parent compound primaquine can inhibit the activity of the P-gp transporter, but has not been shown to inhibit ABCG2 activity [30,45].

The tested compounds exhibited low to moderate cytotoxicity against the cancer model cell lines. The *bis*-urea derivatives **2a** and **2b**, which have a chlorine or trifluoromethyl group, both attached to the benzene ring in the *para* position, were more toxic than the fumardiamides with the same substituents. This suggests that the central group contributes to the toxicity of the compound. The *para*-substituted fumardiamide derivatives **1b** and **1d** showed no toxicity to the same cell line, whereas **1a** with a chlorine atom and **1c** with the trifluoromethyl group attached to the benzene ring in the *meta*-position were more toxic. This indicates the importance of the position of the substituent on the benzene ring. In addition, **1c** and **2a** showed higher toxicity towards the parental PLB-985 than to the PLB/ABCG2 cell line, suggesting that the compounds may be transported by ABCG2 [22].

We investigated the nature of the interaction and the inhibitory potential against ABCG2 for both groups of compounds using various assays. All primaquine derivatives tested decreased the extrusion of the fluorescent dye Hoechst 33342 in MDCKII/ABCG2 and PLB/ABCG2 cell lines via the ABCG2 transporter, suggesting that they interact with the transporter. However, the parent compounds, primaquine and chloroquine, had no effect on the ABCG2-mediated transport of Hoechst 33342. Nevertheless, the specified chemical modifications of the primaquine structure resulted in an inhibitory potential against ABCG2. The initial selection of compounds as potential ABCG2 inhibitors was performed using a binding assay with the conformation-sensitive ABCG2 antibody 5D3. We selected compounds **1a**, **1b**, and **1d** for further analysis. All three compounds inhibited ABCG2-mediated Hoechst transport similarly, based on IC_50_ values ranging from 0.7–1 µM. The maximum inhibition achieved by the tested compounds was 70% for compound **1a**, indicating that they are unable to completely inhibit ABCG2, as has been reported for some inhibitors [46,47]. In addition, primaquine derivatives **1a**, **1b**, and **1d** were found to strongly inhibit the basal ATPase activity of the ABCG2 transporter at low concentrations, confirming their status as potent inhibitors. Finally, low, non-toxic concentrations (0.5 µM) of compounds **1a**, **1b**, and **1d** successfully sensitized the drug-resistant PLB/ABCG2 cell line to the chemotherapeutic agent mitoxantrone, reducing the IC_50_ value by an order of magnitude. These results suggest that these modified antimalarials may have great potential to reverse MDR. ABC transporters have promiscuous drug-binding pockets, which can bind structurally diverse compounds with varying strengths, releasing and pumping them out at different rates. However, the precise nature of these interactions cannot yet be accurately predicted by computer simulations, necessitating experimental validation through various assays [41,48].

To test selectivity towards ABCG2, we examined the effect of primaquine derivatives **1a**, **1b**, and **1d** on P-gp transporter activity. Interestingly, the P-gp (A2780/Adr) expressing cell line showed greater resistance to these compounds than its parental counterpart (A2780), which was more sensitive. This difference in the cytotoxic profile suggests that the compounds may be P-gp substrates. Potent stimulation of the P-gp transporter’s ATPase activity and a lack of significant effects on P-gp-driven calcein efflux confirmed that the new compounds are selective ABCG2 inhibitors at relevant or low concentrations and are less likely to interfere with P-gp function. Based on our results, we can conclude that introducing fumardiamide into the primaquine structure led to a novel biological activity—the inhibition of ABCG2 and the reversal of MDR in vitro. We have demonstrated that fumardiamide derivatives selectively inhibit ABCG2 and are more potent inhibitors than *bis*-ureas with the same side motifs. This emphasizes the importance of the central part of the molecules. These results are consistent with the findings of Moinul et al. regarding the structural features of promising ABCG2 inhibitors [20]. Accordingly, the most important pharmacophoric element of an ABCG2 inhibitor is the fused benzene ring with a six-membered heterocyclic aromatic ring. The nitrogen and oxygen atoms serve as the focal point of the fused heterocyclic ring. Additionally, in some cases, a heterocyclic aromatic ring is connected to a fused ring via a linker, which may be a carbonyl, amino, or amide linker. In our compounds, a fused heterocyclic ring (the primaquine part) is coupled via an amide linker to a halogenated benzene ring (the aromatic part). This structure was found to be essential for activity and selectivity towards the ABCG2 transporter.

In summary, we demonstrated that the biological activity of the original primaquine compound was significantly altered through chemical modification. The newly synthesized primaquine fumardiamide derivatives selectively inhibit ABCG2. These results are of great value for the development of novel selective ABC inhibitors and as potential adjuvant anticancer therapeutics. Although various pharmacokinetic interactions (e.g., interactions with other transporters or metabolizing enzymes) may affect the efficacy of compounds in vivo, the in vitro ABC transporter assays presented here often provide relevant indications of in vivo effects. However, despite primaquine being a clinically approved drug, further toxicity studies will be necessary to evaluate potential changes in toxicity and pharmacokinetics caused by the described chemical modifications. Moreover, comprehensive in vivo experiments are needed to assess the therapeutic potential of these compounds.

## 4. Materials and Methods

### 4.1. Materials

Primaquine fumardiamides with chlorobenzene (**1a** and **1b**) or trifluorobenzene moieties (**1c** and **1d**) and *bis*-ureas (**2a** and **2b**) (Table 1) were prepared according to the protocol described in Rajić et al. [34] and Perković et al. [35]. The chemotherapeutic agent mitoxantrone (MX) and conventional P-gp and ABCG2 inhibitors verapamil and Ko143 were purchased from MilliporeSigma (St. Louis, MO, USA).

### 4.2. Cell Lines

The human acute myeloid leukemia cell line PLB-985, with an overexpression of human ABCG2, was previously prepared via retroviral transduction and selection with the ABCG2 substrate MX [37], whereas the parental PLB-985 cell line was used as a control. Stable transfectants of the Madin–Darby canine kidney cell line (MDCK-II) with the overexpression of the ABCG2 transporter and parental MDCK-II cell line were kindly provided by Dr. A. Schinkel and Dr. P. Borst from the Netherlands Cancer Institute, Amsterdam, Netherlands. The human ovarian carcinoma cell line A2780 (ECACC cat. no. 93112519) and its Adriamycin-resistant counterpart A2780/Adr (ECACC cat. no. 93112520) were obtained from the European Collection of Authenticated Cell Cultures.

Cell lines were cultured in suspension (PLB-985) or as a monolayer (MDCK-II, A2780) in RPMI-1640 (PLB-985) or DMEM (MDCK-II, A2780) culture medium, with the addition of 10% fetal bovine serum (FBS), 2 mM of L-glutamine, 100 U/mL of penicillin, and 100 µg/mL of streptomycin. Cells were kept at 37 °C in a humidified atmosphere with 5% CO_2_.

### 4.3. Cell Proliferation Assay

Cell proliferation and a cytotoxicity assay were performed as previously described by Mioč et al. [49]. Briefly, cells were seeded in a 96-well plate at a concentration of 2.5 × 10^4^ (PLB-985) or 1.5 × 10^3^ (MDCK-II, A2780) cells per well. The next day, the tested compounds were added in serial dilutions (10^−8^–10^−4^ M) alone or at a 5 µM concentration in combination with MX (10^−9^–10^−5^ M). The viability of the cells was evaluated with an MTT assay 72 h after the incubation. The compound concentration at which 50% of cell growth was inhibited (IC_50_) compared with the control cells was calculated for each compound from dose–response curves via a linear regression analysis.

### 4.4. Calcein–AM Accumulation Assay

A calcein-AM (MilliporeSigma, St. Louis, MO, USA) accumulation assay was performed as previously described by Guberović et al. [50]. The cells (A2780) were seeded for 24 h before the experiment in 96-well plates at a concentration of 3 × 10^4^ cells per well. On the day of the experiment, the cells were washed with PBS, and the tested compounds (primaquine derivatives and verapamil) were added at a final concentration of 10 µM in RPMI medium without FBS. Calcein-AM was added at a final concentration of 0.25 µM, and everything was incubated together for 60 min at 37 °C in the dark. The cells were then washed with PBS, and calcein fluorescence was measured using the Tecan Genios microplate reader (Tecan, Männedorf, Switzerland) at excitation/emission wavelengths of 485 nm/530 nm.

### 4.5. Hoechst 33342 Uptake Assay

The Hoechst 33342 (MilliporeSigma, St. Louis, MO, USA) uptake assay was performed as previously described by Mioč et al. [49]. Cells were seeded in 96-well plates at a concentration of 2.5 × 10^4^ cells per well (MDCK-II) or diluted to this concentration on the day of the experiment (PLB-985). On the day of the experiment, the cells were washed with PBS, and the tested compounds were added at a final concentration of 10 µM (primaquine derivatives) or 1 µM (Ko143) in DMEM or RPMI medium without FBS.

For the ABCG2 transporter inhibition assay, the tested compounds were added at a final concentration of 10 µM. The cells were incubated for 5 min before Hoechst 33342 was added at a final concentration of 0.5 µM, and everything was incubated together for 60 min at 37 °C in the dark. The cells were then washed with PBS, and the fluorescence of Hoechst 33342 was measured using the Tecan Genios microplate reader (Tecan, Männedorf, Switzerland) at excitation/emission wavelengths of 360 nm/465 nm.

### 4.6. Membrane Preparations

Membrane vesicles expressing the human ABCG2 transporter were prepared according to the method described by Telbisz et al. [40] and Özvegy-Laczka [38]. Cells of the insect *Spodoptera frugiperda* (Sf9 cell line) were cultured and infected with a recombinant virus carrying the cDNA of human ABCG2 cloned into a baculovirus vector. Insect cells were harvested 72 h after infection, and membranes were isolated through differential centrifugation. The protein concentration was determined using the modified Lowry method. After isolation, membranes were loaded with cholesterol as previously described in Telbisz et al. [40] and membrane fractions were stored at −80 °C.

### 4.7. Protein Expression Analysis (Western Blot)

For protein expression analysis, PLB/ABCG2 cells were harvested after treatment, lysed in RIPA buffer with the addition of protease inhibitor cocktail tablets (Roche, Mannheim, Germany), and sonicated with the Bioruptur sonicator (Diagenode, Danville, NJ, USA). The concentration of total proteins in the cell lysate was determined using the Pierce BCA Protein Assay Kit according to the manufacturer’s instructions (Thermo Fisher Scientific). Twenty micrograms of protein per sample were loaded and separated via SDS-PAGE and then transferred to the PVDF membrane (Roche, Germany). After transfer, the membrane was stained with amido black (0.1% amido black in 10% methanol and 2% acetic acid) to visualize the proteins, which served as a loading control. The membrane was blocked with 5% non-fat milk in Tris-buffered saline with 0.1% Tween (TBST) for 1 h at room temperature and then incubated with the primary anti-BCRP BXP-21 antibody (1:100, IgG2a, Santa Cruz Biotechnology, Dallas, Texas, US, Chicago, IL, USA) at 4 °C overnight. The membrane was washed three times with TBST and incubated with horseradish peroxidase (HRP)–conjugated secondary antibody (anti-mouse IgG-HRP, 1:4000, GE Healthcare) for 2 h at room temperature. After washing three times with TBST, the signal was detected with the Western Lightning Plus-ECL reagent (Perkin-Elmer, Waltham, MA, USA and visualized with the UVITEC imaging system (Cleaver Scientific Ltd., Rugby, UK).

### 4.8. The 5D3 Antibody-Binding Shift Assay

The 5D3 antibody shift assay was performed in the PLB/ABCG2 cell line according to Telbisz et al. [39]. The cells were collected at a concentration of 2 × 10^5^ cells per tube, washed, and resuspended in PBS containing 5.5 mM glucose. First, the cells were incubated with the tested substances (Ko143 at a final concentration of 1 µM or primaquine derivatives at a final concentration of 1, 5, and 10 µM) and 0.5 µg of unlabeled primary monoclonal antibody 5D3 (IgG2bƙ, Millipore, Burlington, MA, USA, catalog number MAB4155) per sample in a blocking solution containing PBS with 5.5 mM glucose and 1% bovine serum albumin (BSA). After incubation at 37 °C for 45 min, the cells were washed twice with ice-cold PBS and incubated with 0.25 µg goat anti-mouse Alexa Fluor 488 secondary antibody (IgG, Thermo Fisher Scientific, Waltham, MA, USA) per sample in a blocking solution containing 0.5% BSA. Cells were incubated for 30 min at 37 °C, then washed with ice-cold PBS and analyzed by flow cytometry (BD FACSCalibur, Becton Dickinson, NJ, USA) with excitation at 488 nm and fluorescence collected through a 530 nm pass filter. Data analysis was performed using FlowJo software. Maximum 5D3 labeling was determined as treatment with 1 µM Ko143 for each sample, as suggested by Özvegy-Laczka et al. [38].

### 4.9. ATPase Assay

The effect of the tested compounds on vanadate-sensitive ATPase activity of ABC transporters was measured in Sf9 cell membranes carrying the human ABCG2 or P-gp transporter via a colorimetric reaction, as described in Telbisz et al. [40]. The tested compounds were prepared in DMSO (the final concentration was a maximum of 1% DMSO). The ABC transporter-related vanadate-sensitive ATPase activity was measured by determining the background in the presence of 1 mM Na-orthovanadate. The reaction mixture consisted of 40 mM MOPS–Tris (pH 7.0), 50 mM KCl, 2 mM dithiothreitol, 0.5 mM EGTA–Tris, 5 mM Na-azide, 1 mM ouabain, and 7.5 µg of membrane protein in a 96-well plate (reaction volume 50 μL). ATPase reaction activity was initiated via the addition of 3.3 mM MgATP. As reference compounds, quercetin (stimulatory substrate) and Ko143 (inhibitor) were used for ABCG2, and verapamil (stimulatory substrate) was used for P-gp. The reaction was stopped by adding the colorimetric reaction compounds to wells: 50μL of reagent P (containing 2.5 M H_2_SO_4_, 1% ammonium molybdate, and 0.014% antimony potassium tartarate), 30 μL of 20% acetic acid, and 25 μL of 1% ascorbic acid. After 25 min of incubation, the optical density was measured at 660 nm in VictorX3 plate reader (Perkin-Elmer).

### 4.10. Immunofluorescence

For the immunofluorescence studies, cells (MDCK-II/ABCG2) were prepared as described by Mioč et al. [49]. Glass slides were pretreated overnight with FBS at 37 °C before the cells were seeded and allowed to adhere. The next day, the cells were treated with 5 µM of the tested substances, and the cellular ABCG2 protein was stained 72 h after treatment with the primary antibody BXP-21 (1:100, IgG2a, Santa Cruz Biotechnology, Dallas, TX, USA, catalog number sc-58222). Slides were fixed with 4% paraformaldehyde (PFA) for 10 min and permeabilized with 0.1% saponin in PBS with Ca/Mg for another 10 min. Slides were washed in PBS with Ca/Mg, blocked in blocking solution (1% BSA and 0.1% saponin in PBS with Ca/Mg) for 1 h at room temperature, and incubated with the primary antibody BXP-21 overnight at 4 °C. Slides were washed and incubated with the secondary antibody goat anti-mouse Alexa Fluor 488 (1:250, Thermo Fisher Scientific, Waltham, MA, USA) in a blocking solution for 1 h at room temperature. After incubation, slides were washed and incubated with DAPI (1 µg/mL) for 2 min at room temperature, washed thoroughly, and mounted with Fluoromount antifade reagent (MilliporeSigma, St. Louis, MO, USA). Blue (461 nm) and green (505–525 nm) fluorescence was recorded using a Leica SP8 confocal microscope (TCS X, Leica Microsystems GmbH, Wetzlar, Germany) at 405 nm and 488 nm excitation, respectively. Image depth: 8-bit; objective specifications: HC PL APO CS2 63×/1.40 oil objective; resolution: 1744 × 1744; pixel size (xy): 0.071 × 0.071 µm; gain–Hoechst 33342—hybrid detector set to 500, GFP hybrid detector set to 170, gating 0.65–6 ns. The images were created using the Adobe Illustrator CS2 software.

### 4.11. Real-Time Hoechst 33342 Uptake Study

To monitor the uptake of Hoechst 33342 in real time, the HEK-293 cell line was transfected with a vector containing the EGFP-tagged ABCG2 protein (pEGFP-G2), kindly provided by Laszlo Homolya from the Institute of Enzymology, Research Center for Natural Sciences, Budapest, Hungary. The study was performed as described previously by Orbán et al. [36]. In brief, cells were seeded in four-chamber 35 mm glass-bottom dishes (Cellvis, Mountain View, CA, US in DMEM medium without antibiotics at a density of 10^5^ cells per well. The cells were transfected with pEGFP-G2 using Lipofectamine 2000 (Thermo Fisher Scientific, Waltham, MA, USA) according to the manufacturer’s instructions. The medium was changed 4 h after transfection, and the cells were incubated for an additional 48 h before observation. For the real-time fluorescence study, 8 µM of Hoechst 33342 was added to the transfected cells in an FBS-free medium. Blue (461 nm) and green (505–525) fluorescence was recorded simultaneously with a confocal microscope at 405 and 488 nm excitation, respectively (confocal laser scanning microscope TCS X, Leica Microsystems GmbH, Wetzlar, Germany) for 7 min. To block the extrusion of the dye via the ABCG2 transporter, Ko143 (1 µM) or the tested compounds (5 µM) were added, and fluorescence was recorded for 5 min. Image depth: 8-bit; objective lens specifications: HC PL APO CS2 63×/1.40 oil objective; resolution: 1024 × 1024; pixel size (xy): 0.18 × 0.18 µm, 1 min 15.81 s; gain: Hoechst 33342 hybrid detector set to 178–500 (changed during samples according to Hoechst intensity in each sample), GFP hybrid detector set to 238, gating 0.63–6 ns.

For the quantification of the images, the cells were divided into non-transfected and transfected cells based on green fluorescence. For each condition (and experiment), at least 50 cells were analyzed in four to six positions per time point (0, 2, 5, 7, and 9 min) in two individual experiments to monitor the kinetics of intracellular drug accumulation. Blue fluorescence was monitored in regions of interest (ROIs) positioned in the nucleus. The Leica Application Suite X software (LAS X, 3.1.1.15751, Leica Microsystems GmbH, Wetzlar, Germany) was used for image acquisition. ImageJ software Version 1.54g was used for image analysis and quantification, and Adobe Illustrator CS2 was used for image preparation.

### 4.12. Statistics

The data are presented graphically as means ± standard deviations (SD). A one-way ANOVA with Dunnett’s post hoc test was used to determine statistical significance. Statistical calculations were performed in GraphPad Prism 5 (NS: not significant; * *p* < 0.05, ** *p* < 0.01, and *** *p* < 0.001). K_m_ and K_i_ values of ATPase activity (Michaelis–Menten constants) were determined from Hill1 non-linear curve fitting in OriginPro8 software.

## Figures and Tables

**Table 1:** Chemical structures and antiproliferative activity of studied primaquine fumardiamide (**1a**–**d**) and *bis*-urea derivatives (**2a**, **b**) in PLB-985 and PLB/ABCG2 cell lines. IC_50_ indicates the concentration of the compound that causes half-maximal inhibition of cell growth. Data represent the mean ± SD of three independent experiments done in quadruplicate.

Compd.	Structure	IC_50_ (µM)	
		PLB-985	PLB/ABCG2
**1a**	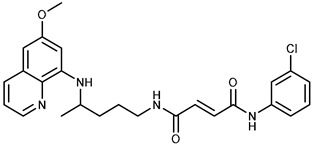	37.7 ± 0.7	68.6 ± 9.8
**1b**	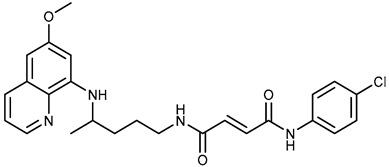	≥100	≥100
**1c**	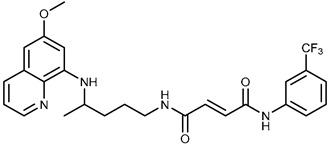	47.7 ± 14.9	≥100
**1d**	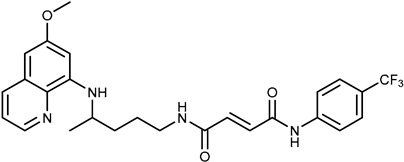	≥100	≥100
**2a**	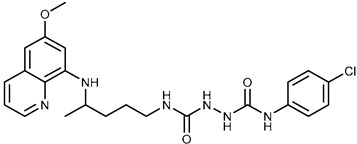	43.8 ± 5.3	≥100
**2b**	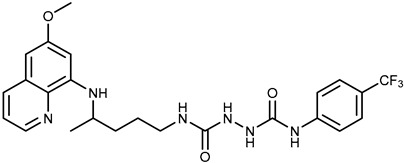	9.37 ± 1.09	12.1 ± 6.1

**Table 2:** Growth inhibition of PLB-985 and PLB/ABCG2 cell lines with mitoxantrone (MX) alone or in combination with tested compounds or Ko143. IC_50_ values were calculated from cell viability curves plotted from three independent experiments done in quadruplicate.

Compd.	IC_50_ (μM)	
	PLB-985	PLB/ABCG2
Mitoxantrone (MX)	0.003 ± 0.0001	0.12 ± 0.0002
MX + Ko143 (1 µM)	0.003 ± 0.0008	0.01 ± 0.004
MX + **1a** (0.5 µM)	0.0021 ± 0.0015	0.01 ± 0.005
MX + **1b** (0.5 µM)	0.003 ± 0.0007	0.02 ± 0.009
MX + **1d** (0.5 µM)	0.0028 ± 0.001	0.01 ± 0.006

**Table 3:** Antiproliferative activity of primaquine and halogenaniline fumardiamides **1a**, **1b**, and **1d** in A2780 and A2780/Adr (P-gp expressing) cell lines. IC_50_ indicates the concentration of the compound that causes half-maximal inhibition of cell growth. Data represent the means ± SDs of three independent experiments performed in quadruplicate.

Compd.	IC_50_ (μM)	
	A2780	A2780/Adr
**1a**	6.7 ± 0.4	14 ± 0.5
**1b**	≥100	≥100
**1d**	50 ± 0.2	≥100

## Data Availability

The data presented in this study are available upon request to interested researchers.

## Abbreviations

The following abbreviations are used in this manuscript:

ABC	ATP-binding cassette
BCRP	breast cancer resistance protein
BSA	bovine serum albumin
CSCs	cancer stem cells
DMEM	Dulbecco’s Modified Eagle Medium
FBS	fetal bovine serum
MDR	multidrug resistance
MTT	3-(4,5-dimethylthiazol-2-yl)-2,5-diphenyl-2H-tetrazolium bromide
MX	mitoxantrone
P-gp	P-glycoprotein
SDS-PAGE	sodium dodecyl sulfate polyacrylamide gel electrophoresis
pEGFP-G2	vector containing the EGFP-tagged ABCG2
VER	verapamil
